# Comparison of ventilation defects quantified by Technegas SPECT and hyperpolarized ^129^Xe MRI

**DOI:** 10.3389/fphys.2023.1133334

**Published:** 2023-04-28

**Authors:** Nisarg Radadia, Yonni Friedlander, Eldar Priel, Norman B. Konyer, Chynna Huang, Mobin Jamal, Troy Farncombe, Christopher Marriott, Christian Finley, John Agzarian, Myrna Dolovich, Michael D. Noseworthy, Parameswaran Nair, Yaron Shargall, Sarah Svenningsen

**Affiliations:** ^1^ Division of Respirology, Department of Medicine, McMaster University, Hamilton, ON, Canada; ^2^ Firestone Institute for Respiratory Health, St. Joseph’s Healthcare Hamilton, Hamilton, ON, Canada; ^3^ Imaging Research Centre, St. Joseph’s Healthcare Hamilton, Hamilton, ON, Canada; ^4^ Division of Thoracic Surgery, St. Joseph’s Healthcare Hamilton, Hamilton, ON, Canada; ^5^ Division of Thoracic Surgery, Department of Surgery, McMaster University, Hamilton, ON, Canada; ^6^ Department of Radiology, McMaster University, Hamilton, ON, Canada; ^7^ Department of Nuclear Medicine, St. Joseph’s Healthcare Hamilton, Hamilton, ON, Canada; ^8^ Department of Electrical and Computer Engineering, McMaster University, Hamilton, ON, Canada

**Keywords:** ventilation imaging, Technegas SPECT, hyperpolarized xenon-129, MRI, airflow obstruction, ventilation defects, functional lung imaging

## Abstract

**Introduction:** The ideal contrast agents for ventilation SPECT and MRI are Technegas and ^129^Xe gas, respectively. Despite increasing interest in the clinical utility of ventilation imaging, these modalities have not been directly compared. Therefore, our objective was to compare the ventilation defect percent (VDP) assessed by Technegas SPECT and hyperpolarized ^129^Xe MRI in patients scheduled to undergo lung cancer resection with and without pre-existing obstructive lung disease.

**Methods:** Forty-one adults scheduled to undergo lung cancer resection performed same-day Technegas SPECT, hyperpolarized ^129^Xe MRI, spirometry, and diffusing capacity of the lung for carbon monoxide (DL_CO_). Ventilation abnormalities were quantified as the VDP using two different methods: adaptive thresholding (VDP_T_) and k-means clustering (VDP_K_). Correlation and agreement between VDP quantified by Technegas SPECT and ^129^Xe MRI were determined by Spearman correlation and Bland-Altman analysis, respectively.

**Results:** VDP measured by Technegas SPECT and ^129^Xe MRI were correlated (VDP_T_: r = 0.48, *p* = 0.001; VDP_K_: r = 0.63, *p* < 0.0001). A 2.0% and 1.6% bias towards higher Technegas SPECT VDP was measured using the adaptive threshold method (VDP_T_: 23.0% ± 14.0% vs. 21.0% ± 5.2%, *p* = 0.81) and k-means method (VDP_K_: 9.4% ± 9.4% vs. 7.8% ± 10.0%, *p* = 0.02), respectively. For both modalities, higher VDP was correlated with lower FEV_1_/FVC (SPECT VDP_T_: r = −0.38, *p* = 0.01; MRI VDP_K_: r = −0.46, *p* = 0.002) and DL_CO_ (SPECT VDP_T_: r = −0.61, *p* < 0.0001; MRI VDP_K_: r = −0.68, *p* < 0.0001). Subgroup analysis revealed that VDP measured by both modalities was significantly higher for participants with COPD (n = 13) than those with asthma (n = 6; SPECT VDP_T_: *p* = 0.007, MRI VDP_K_: *p* = 0.006) and those with no history of obstructive lung disease (n = 21; SPECT VDP_T_: *p* = 0.0003, MRI VDP_K_: *p* = 0.0003).

**Discussion:** The burden of ventilation defects quantified by Technegas SPECT and ^129^Xe MRI VDP was correlated and greater in participants with COPD when compared to those without. Our observations indicate that, despite substantial differences between the imaging modalities, quantitative assessment of ventilation defects by Technegas SPECT and ^129^Xe MRI is comparable.

## 1 Introduction

Pulmonary ventilation imaging modalities have been developed to provide a regional evaluation of airflow obstruction at high-resolution and thus ultimately improve the clinical management of a variety of lung diseases. Nuclear medicine (Jögi et al., 2011; Bajc et al., 2017; Farrow et al., 2017), magnetic resonance imaging (MRI) (Kruger et al., 2016; Ohno et al., 2022; Sharma et al., 2022) and computed tomography (CT) (Park et al., 2010; Kim et al., 2012) based methods have all demonstrated abnormal and heterogeneous ventilation in patients with obstructive lung diseases, including chronic obstructive pulmonary disease (COPD) and asthma. While the potential added value of ventilation imaging modalities over conventional global measures of lung function made by breathing tests is recognized, few are widely available or used in the routine management of obstructive lung disease.

The most clinically established and widely used ventilation imaging modality is single photon emission computed tomography (SPECT) using a range of ventilation agents including krypton-81 m gas (^81m^Kr) and ^99m^Tc-labelled aerosols (e.g., diethylene-triamine-pentaacetate [DTPA] and Technegas) (Roach et al., 2013; Bajc et al., 2019). Beyond its primary use in conjunction with perfusion SPECT for the diagnosis of pulmonary embolism, ventilation SPECT is rarely utilized for other indications such as pre-operative quantification of lung function (Genseke et al., 2018) and functional lung avoidance in radiation therapy planning (Munawar et al., 2010; Yuan et al., 2011). Alternatively, inhaled hyperpolarized gas MRI, using either helium-3 (^3^He) or xenon-129 (^129^Xe), has undergone extensive research and development for obstructive lung disease applications (Kirby et al., 2012; Svenningsen et al., 2013; Ebner et al., 2017; Shammi et al., 2022; Stewart et al., 2022). Compared to SPECT, hyperpolarized gas MRI offers higher spatial and temporal resolution without exposure to ionizing radiation. However, its availability is currently limited to specialized academic centers. Previous cross-modality investigations have demonstrated the comparability of ^81m^Kr SPECT with ^3^He MRI in 23 patients with COPD and 9 healthy volunteers (Stavngaard et al., 2005), and ^99m^Tc-DTPA SPECT with ^129^Xe MRI in 11 patients with COPD (Doganay et al., 2019; Kim et al., 2019). While these preliminary investigations report good comparability, they were limited by the small number of patients and disease populations evaluated. Most importantly, the current ideal contrast agents for ventilation SPECT and MRI are generally accepted to be Technegas (Bajc et al., 2019) and ^129^Xe gas (Niedbalski et al., 2021), respectively, and they have not been directly compared to each other.

With broadening interest in the clinical utility of ventilation imaging, and recent approval of ^129^Xe MRI and impending approval of Technegas SPECT by the U.S. Food and Drug Administration, a direct quantitative comparison of the modalities is needed. Therefore, the primary objective of this study was to compare the ventilation defect percent (VDP) assessed by Technegas SPECT and ^129^Xe MRI obtained the same day in a convenient sample of patients scheduled to undergo lung cancer resection with and without pre-existing obstructive lung disease. The secondary objective was to evaluate and compare the relationship of VDP assessed by both modalities with clinical history and standard lung function measures of obstructive lung diseases. To address these objectives, ventilation defects observed by Technegas SPECT and ^129^Xe MRI were quantified as the whole-lung VDP using two previously published segmentation methods: adaptive thresholding (VDP_T_), previously optimized for Technegas SPECT (Farrow et al., 2012); and, k-means clustering (VDP_K_), previously optimized for ^129^Xe MRI (Kirby et al., 2012).

## 2 Materials and methods

### 2.1 Participants and study design

This was a prospectively planned sub-study of patients scheduled to undergo first-time lung cancer resection at the division of Thoracic Surgery, McMaster University, Hamilton, Ontario as part of their clinical care who were enrolled into a single-center, prospective, 5-week observational study designed to evaluate the prevalence and clinical relevance of abnormal ventilation in lung cancer patients prior to lung resection. Eligible patients were greater than 18 years of age, first-time lung resection candidates in accordance with the British Thoracic Society guidelines (Callister et al., 2015), and they could not have had previous lung resection, previous chest radiation, or MRI contraindications. All participants provided written informed consent to an ethics-board approved (Hamilton Integrated Research Ethics Board #7770) and registered (ClinicalTrials.gov #NCT04191174) protocol. We report data acquired at a single pre-operative study visit, at which time baseline demographic data and clinical history were collected, and participants performed standard-of-care pulmonary function testing (spirometry and diffusing capacity of the lung for carbon monoxide (DL_CO_)), Technegas SPECT-CT and ^129^Xe MRI. Image session order was randomized.

### 2.2 Technegas SPECT-CT acquisition

Technegas (Cyclomedica Australia, Sydney) was prepared with a Technegas Generator (Cyclomedica Australia, Sydney) according to the manufacturer recommendations and a 40 MBq dose was administered to the participant in the supine position via inhalation. The participant was coached to inhale Technegas, starting at functional residual capacity, until 40 μSv/h was measured by a hand-held Geiger counter positioned over the chest. Technegas SPECT was then acquired while supine, during 15-min of tidal breathing using an Optima™ Nuclear Medicine (NM)/Computed Tomography (CT) 640 hybrid imaging system (GE Healthcare, Milwaukee, United States) and in accordance with The Canadian Association of Nuclear Medicine guidelines using the following acquisition parameters: LEHR collimator, energy window: 140 keV ± 20%, zoom factor of 1.0, 128 × 128 matrix and 4.42 mm isotropic voxels, step and shoot, 25 s/image, 60 images per acquisition (30 images per camera head), 360° rotation, 6° steps, body contour. A low dose non-contrast chest CT was subsequently acquired on the same NM/CT system during free breathing for attenuation correction and to allow for delineation of the thoracic cavity volume using the following acquisition and reconstruction parameters: 120 kVp, 20 mA, 1 s tube rotation time, 1.25 pitch, 512 × 512 matrix, 2.5 mm slice thickness, 2.5 mm slice spacing, standard reconstruction kernel, and 50 cm display field of view. Technegas SPECT reconstruction was performed using a Hermes Workstation (Hermes Medical Solutions, Stockholm, Sweden) with the following settings: OSEM reconstruction (2 iterations, 10 subsets), 3D Gaussian filter with 1.20 cm FWHM with corrections for attenuation, scatter, and collimator resolution recovery.

### 2.3 MRI acquisition

^129^Xe ventilation MRI and ^1^H MRI were acquired using a Discovery™ MR750 3T system (General Electric Healthcare; Milwaukee, United States) as previously described (Svenningsen et al., 2021). Participants were instructed to inhale 1 L of gas (N_2_ for ^1^H MRI and a hyperpolarized ^129^Xe/N_2_ mixture for ^129^Xe MRI) from functional residual capacity, and coronal slices were acquired under breath-hold conditions. Spin-exchange polarizer systems (Polarean 9800 or 9820, Polarean, Durham, United States) were used to polarize isotopically enriched ^129^Xe gas (86%; ∼600 mL) that was dispensed into a pre-filled mixing syringe (∼400 mL of N_2_) to achieve a fixed dose of 1 L that was transferred to a Tedlar bag (Jensen Inert Products, Coral Springs, United States) for participant delivery. ^129^Xe polarization was measured using a polarization measurement station (Polarean Inc., Durham, United States) and the dose-equivalent (DE) volume of 100% enriched, 100% polarized ^129^Xe was calculated as previously described (He et al., 2015). Following inhalation of the 1 L dose from functional residual capacity, hyperpolarized ^129^Xe static ventilation MRI was performed using a custom-built, unshielded quadrature-asymmetric bird-cage coil and a 3D fast gradient recalled echo sequence (acquisition time = 10 s, TE = 1.5 ms, TR = 5.1 ms, variable flip angle, initial flip angle = 1.3°, receive bandwidth = 16 kHz, field of view = 40 × 40 × 24 cm^3^, reconstructed matrix size = 128 × 128 × 16, voxel size = 3.125 × 3.125 × 15 mm^3^). A matching ^1^H MRI was performed using the whole-body radiofrequency coil and a fast-spoiled gradient echo sequence (acquisition time = 9 s, TE = 1.2 ms, TR = 4.3 ms, flip angle = 20^°^, FOV = 40 × 40 cm, matrix size = 128 × 128, 16 slices, voxel size = 3.125 × 3.125 × 15 mm^3^).

### 2.4 VDP quantification

Ventilation defects observed by Technegas SPECT and ^129^Xe MRI were quantified as the whole-lung VDP using two different segmentation methods: adaptive thresholding (VDP_T_) and k-means clustering (VDP_K_), which have been optimized and validated for Technegas SPECT (Farrow et al., 2012) and ^129^Xe MRI (Kirby et al., 2012), respectively. For the adaptive thresholding method (Farrow et al., 2012), voxels within the thoracic cavity were defined as “ventilation defect” if they were below a threshold determined as 0.5 x Mean_5-80_, where Mean_5-80_ is the mean intensity of all voxels in the thoracic cavity that fall between the 5th and 80th percentile of voxel intensities. The k-means method (Kirby et al., 2012) used an iterative algorithm to bin the voxel intensities into five clusters, with the lowest signal cluster being considered “ventilation defect.” For both segmentation methods, the whole-lung VDP was calculated as the volume of ventilation defects normalized to the thoracic cavity volume.

#### 2.4.1 SPECT segmentation

The thoracic cavity was delineated by registering the CT to the Technegas SPECT and then segmenting the CT using semi-automated segmentation and registration software implemented on a HERMES workstation. Technegas SPECT ventilation segmentation using the threshold method was implemented on a HERMES workstation and the k-means method was implemented using the Image Processing Toolbox provided by MATLAB R2022b (The MathWorks Inc., Natick, MA, United States).

#### 2.4.2 MRI segmentation

The thoracic cavity was delineated by registering the ^1^H MRI to the ^129^Xe MRI and then segmenting the ^1^H MRI using a previously described semi-automated pipeline implemented in MATLAB (Kirby et al., 2012). ^129^Xe MRI ventilation segmentation using the threshold method was implemented in MATLAB and the k-means method was performed using the previously described MATLAB pipeline (Kirby et al., 2012).

### 2.5 Statistical analysis

Data were tested for normality using the Shapiro-Wilk normality test and, when data were not normal, non-parametric tests were performed. Differences in demographic and clinical characteristics between participants with no history of lung disease, asthma, and COPD were determined using a one-way ANOVA with Tukey’s multiple comparisons test for parametric data or Kruskal Wallis with Dunn’s multiple comparisons test for non-parametric data. The correlation and agreement between VDP measured by Technegas SPECT and ^129^Xe MRI were evaluated by Spearman (ρ) correlation coefficients and Bland-Altman analysis, respectively. The relationship of VDP measured by Technegas SPECT and ^129^Xe MRI with age, pack-year smoking history, spirometry, and DL_CO_ were evaluated by Pearson (r) or Spearman (ρ) correlation coefficients. Statistical analyses were performed using GraphPad Prism 8.0 (GraphPad Software, San Diego, CA, United States) and all results were considered significant when the probability of making a Type I error was less than 5% (*p* < 0.05).

## 3 Results

Forty-four patients scheduled for resection of lung cancer were enrolled, and 41 who completed same-day Technegas SPECT and ^129^Xe MRI were included in our analysis. Three of the enrolled participants were excluded from our analysis because ^129^Xe MRI was not performed; two participants had an MRI contraindication (brain aneurism clip), and one was unable to accommodate MRI scheduling. Of the 41 participants evaluated, 21 (51%) had no concomitant history of lung disease, while 6 (15%) had a history of asthma, 13 (32%) had COPD, and 1 (2%) had interstitial lung disease (ILD). Participant demographics, clinical characteristics, and primary tumor characteristics are summarized in Table 1. Participants with no history of lung disease, asthma, and COPD were well-balanced with respect to age (*p* = 0.41) and BMI (*p* = 0.08). Participants with COPD had a higher pack-year smoking history and lower DL_CO_%_pred_ than participants with asthma (*p* = 0.01 and *p* = 0.0004) and those with no history of lung disease (*p* = 0.02 and *p* < 0.0001). FEV_1_%_pred_ and FEV_1_/FVC were also lower for participants with COPD than those with no history of lung disease (*p* = 0.005 and *p* = 0.002).

**TABLE 1:** Participant demographics and clinical characteristics.

		History of obstructive lung disease			
	All (n = 41)	None (n = 21)	Asthma (n = 6)	COPD (n = 13)	*p*-value*
Age years	68 ± 7	69 ± 9	64 ± 6	68 ± 6	0.41
Female sex n (%)	25 (61)	14 (67)	5 (83)	6 (46)	--
BMI kg/m^2^	27 ± 6	27 ± 6	31 ± 6	24 ± 7	0.08
Smoking history					
Never n (%)	8 (20)	7 (33)	1 (17)	0 (0)	--
Past n (%)	24 (59)	10 (48)	5 (83)	8 (62)	--
Current n (%)	9 (22)	4(19)	0 (0)	5 (38)	--
Pack-years	27[0–100]	20[0–66]	9[0–25]	50[14–100]	**0.005** ^†^
Pulmonary function tests					
FEV_1_%_pred_	84 ± 23	94 ± 19	83 ± 16	69 ± 27	**0.007** ^‡^
FVC%_pred_	98 ± 18	104 ± 20	97 ± 12	92 ± 15	0.17
FEV_1_/FVC %	67 ± 13	72 ± 10	69 ± 6	57 ± 16	**0.003** ^‡^
DL_CO_%_pred_	93 ± 31	107 ± 24	111 ± 24	62 ± 22	**<0.0001** ^†^
Primary tumor characteristics					
NSCLC n (%)	33 (80)	16 (76)	4 (66)	12 (92)	--
SCLC n (%)	1 (2)	--	1 (17)	--	--
Other n (%)	3 (7)	2 (10)	1 (17)	--	--
Stage n (%)^#^					
Stage I	25 (61)	12 (57)	5 (83)	8 (62)	--
Stage II	7 (17)	4 (19)	--	2 (15)	--
Stage III	3 (7)	--	1 (17)	2 (15)	--
Stage IV	2 (5)	2 (10)	--	--	--
T-classification n (%)^#^					
T_1_ (≤3 cm)	19 (46)	8 (38)	3 (50)	8 (62)	--
T_2_ (>3 to ≤5 cm)	11 (27)	8 (38)	2 (33)	1 (8)	--
T_3_ (>5 to ≤7 cm)	4 (10)	1 (5)	1 (17)	1 (8)	--
T_4_ (>7 cm)	3 (7)	1 (5)	--	2 (15)	--

Values are mean ± standard deviation or median [minimum-maximum] except when indicated otherwise. BMI = body mass index; COPD = chronic obstructive pulmonary disease; FEV_1_ = forced expiratory volume in one second; FVC = forced vital capacity; DL_CO_ = diffusion capacity for carbon monoxide; NSCLC = non-small cell lung cancer; SCLC = small cell lung cancer; %_pred_ = percent of predicted value. ^#^As per TNM-staging 8^th^ edition. *Significance of difference between groups was determined using a one-way ANOVA, with Tukey’s multiple comparisons test (parametric data) or Kruskal Wallis with Dunn’s multiple comparisons test (non-parametric data). Multiple comparisons revealed ^†^COPD, different from asthma and none, ^‡^COPD, different from none. Bold values denote statistical significance at the *p* < 0.05 level.

**FIGURE 1:**
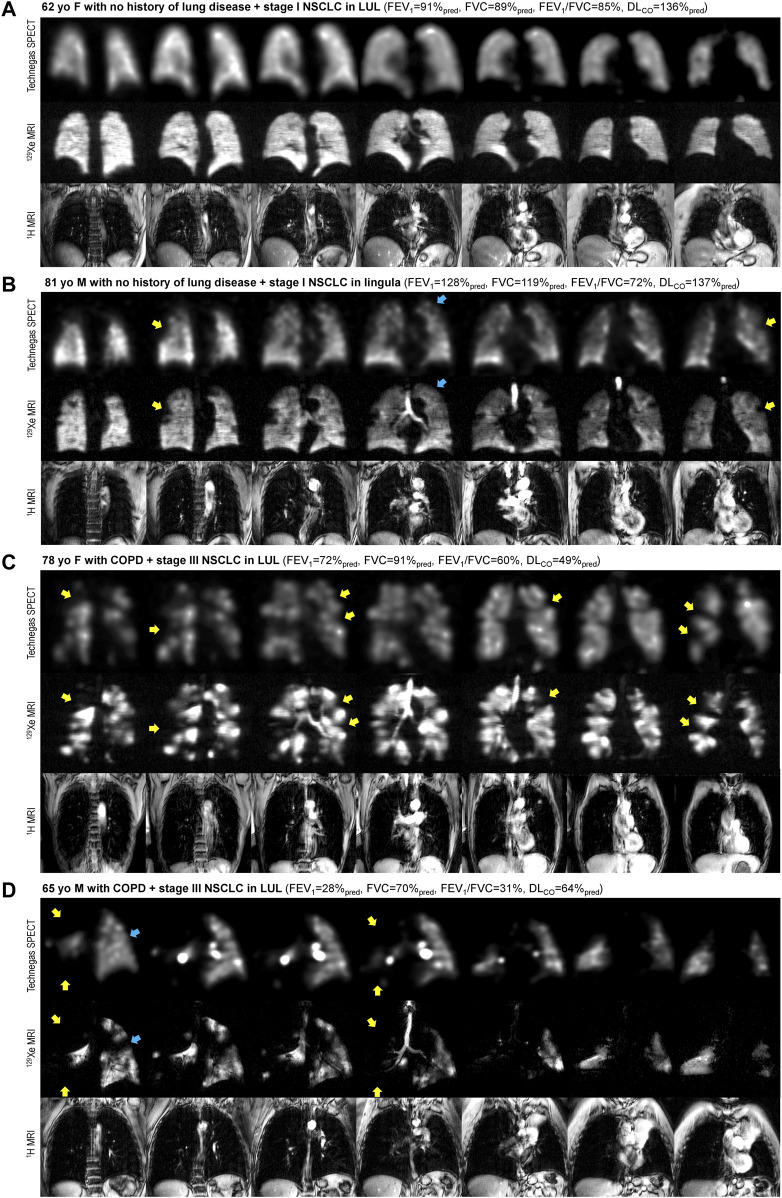
Comparison of ventilation visualized by Technegas SPECT and hyperpolarized ^129^Xe MRI. Anatomically matched coronal Technegas SPECT, ^129^Xe MRI, and ^1^H MRI slices for four representative participants. Select examples of spatially concordant and discordant ventilation defects are highlighted by yellow and blue arrows, respectively. **(A)** 62-year-old female with necrotizing granuloma in left upper lobe and no history of lung disease. Technegas SPECT: VDP_T_ = 12%*, VDP_K_ = 5%; ^129^Xe MRI: VDP_T_ = 22%, VDP_K_ = 1%*; FEV_1_ = 91%_pred_, FVC = 89%_pred_, FEV_1_/FVC = 85%, DL_co_ = 136%_pred_. **(B)** 81-year-old male with stage I NSCLC in lingula and no history of lung disease. Technegas SPECT: VDP_T_ = 28%*, VDP_K_ = 14%; ^129^Xe MRI: VDP_T_ = 18%, VDP_K_ = 4%*; FEV_1_ = 128%_pred_, FVC = 119%_pred_, FEV_1_/FVC = 72%, DL_CO_ = 137%_pred_. **(C)** 78-year-old female with stage III NSCLC in left upper lobe and concomitant COPD. Technegas SPECT: VDP_T_ = 47%*, VDP_K_ = 20%; ^129^Xe MRI: VDP_T_ = 31%, VDP_K_ = 19%*; FEV_1_ = 72%_pred_, FVC = 91%_pred_, FEV_1_/FVC = 60%, DL_CO_ = 49%_pred_. **(D)** 65-year-old male with stage III NSCLC in left upper lobe and concomitant COPD. Technegas SPECT: VDP_T_ = 84%*, VDP_K_ = 52%; ^129^Xe MRI: VDP_T_ = 37%, VDP_K_ = 48%*; FEV_1_ = 28%_pred_, FVC = 70%_pred_, FEV_1_/FVC = 31%, DL_CO_ = 64%_pred_. COPD = chronic obstructive pulmonary disease; FEV_1_ = forced expiratory volume in one second; FVC = forced vital capacity; DL_CO_ = diffusing capacity for carbon monoxide; LUL = left upper lobe; NSCLC = non-small cell lung cancer; %_pred_ = percent of predicted value. *Thresholding (VDP_T_) and k-means clustering (VDP_K_) methods previously optimized and validated for Technegas SPECT and ^129^Xe MRI, respectively.

Technegas SPECT and ^129^Xe MRI were well-tolerated by all participants, with no occurrence of adverse events. The scanning sessions were performed 90 ± 30 min apart [minimum of 12 min, maximum of 120 min]. Dosing and measurements of image quality are provided in the online supplement (Supplementary Table S1). Figure 1 shows coronal Technegas SPECT, ^129^Xe MRI, and corresponding structural ^1^H MRI slices for four representative participants. For participant A, a never-smoker with no history of lung disease, both modalities revealed relatively normal ventilation. For participant B, an ex-smoker with no history of lung disease, both modalities revealed peripheral ventilation defects despite normal lung function assessed by spirometry. For participants C and D, past smokers with COPD, large and spatially concordant ventilation defects were observed by both modalities. While most ventilation defects, such as those highlighted by yellow arrows, were spatially concordant across modalities, focal discordances were also observed, such as those highlighted by blue arrows for participants B and D.

**FIGURE 2:**
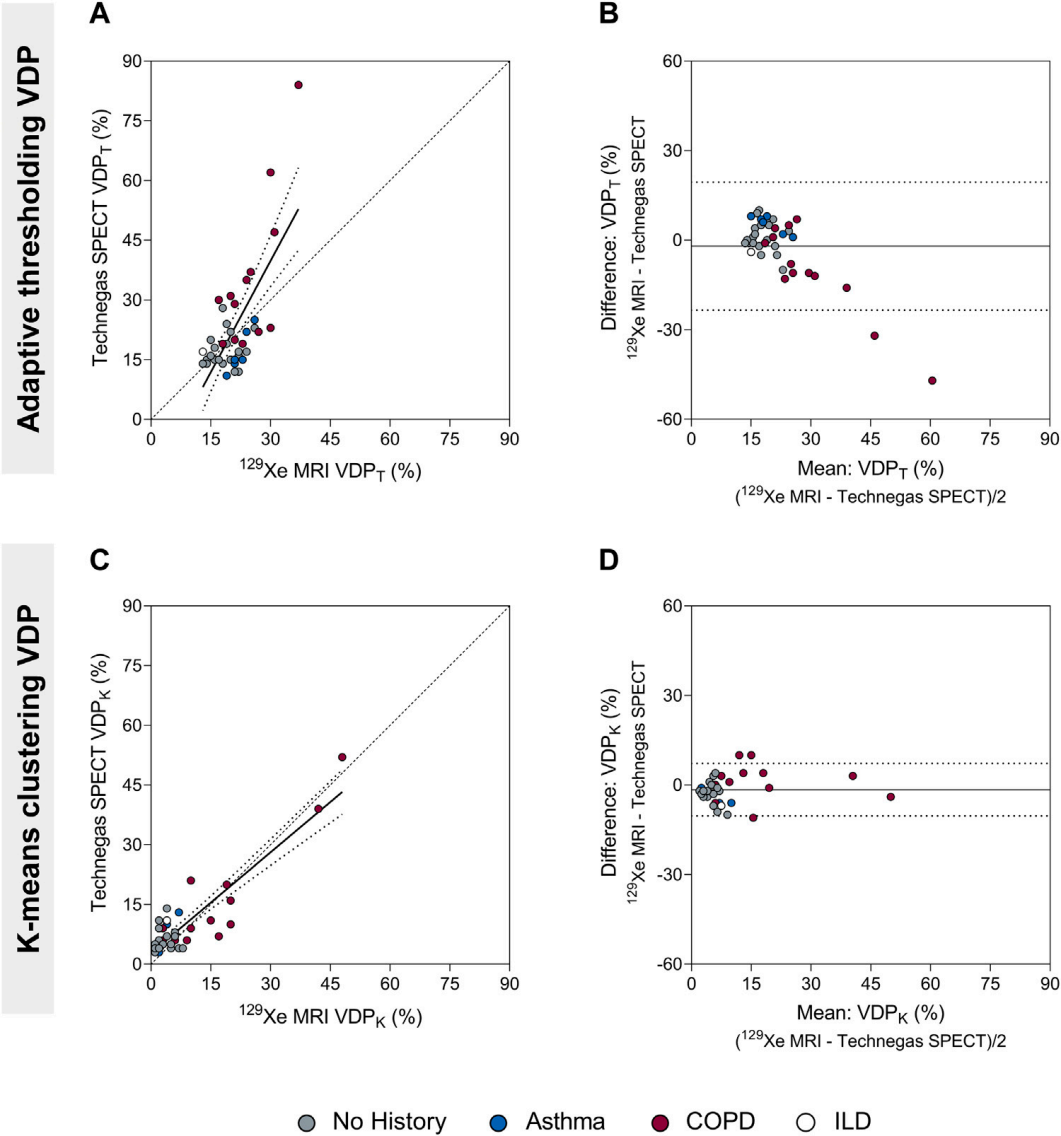
Comparison of the ventilation defect percent (adaptive thresholding (VDP_T_) and k-means clustering (VDP_K_)) quantified by Technegas SPECT and ^129^Xe MRI. **(A)** Positive relationship between Technegas SPECT and ^129^Xe MRI VDP_T_ quantified using the adaptive threshold method (r = 0.48, *r* ^2^ = 0.49, *p* = 0.001, y = 1.86x-16.11). **(B)** Bland-Altman plot of the difference between Technegas SPECT and ^129^Xe MRI VDP_T_ quantified using the adaptive threshold method. Bias = −2.0% (95% limits of agreement, −23.4% to 19.4%). **(C)** Positive relationship between Technegas SPECT and ^129^Xe MRI VDP_K_ quantified using the k-means clustering method (r = 0.63, *r* ^2^ = 0.80, *p* < 0.0001, y = 0.84x+2.83). **(D)** Bland-Altman plot of the difference between Technegas SPECT and ^129^Xe MRI VDP_K_ quantified using the k-means clustering method. Bias = −1.6% (95% limits of agreement, −10.4% to 7.2%). For correlation plots, the dashed line represents the line of identity (y = x) and the dotted lines represent the 95% confidence intervals of the linear regression line. For Bland-Altman plots, the solid line represents the mean of the paired differences, and the dotted lines represent the 95% limits of agreement. Colored data points represent history of lung disease (no history, n = 21; asthma, n = 6; COPD, n = 13; ILD: n = 1). *Thresholding (VDP_T_) and k-means clustering (VDP_K_) methods previously optimized and validated for Technegas SPECT and ^129^Xe MRI, respectively.

The VDP for Technegas SPECT and ^129^Xe MRI were quantified using adaptive thresholding (VDP_T_) and k-means clustering (VDP_K_) methods. The VDP was higher when determined using the threshold method compared to the k-means method for both Technegas SPECT (VDP_T_ = 23.0 ± 14.0% vs. VDP_K_ = 9.4 ± 9.4%, *p* < 0.0001) and ^129^Xe MRI (VDP_T_ = 21.0 ± 5.2% vs. VDP_K_ = 7.8 ± 10.0%, *p* < 0.0001). Figure 2 summarizes the correlation and agreement of VDP_T_ and VDP_K_ measured by Technegas SPECT and ^129^Xe MRI. For both quantification methods, VDP measured by Technegas SPECT and ^129^Xe MRI were correlated (Figure 2A: VDP_T_, r = 0.48, *p* = 0.001; Figure 2C: VDP_K_, r = 0.63, *p* < 0.0001). Using the threshold method, Bland-Altman analysis (Figure 2B) indicated a 2.0% bias (95% limit of agreement: −23.4% to 19.4%) for higher VDP_T_ measured by Technegas SPECT (Technegas SPECT VDP_T_ = 23.0 ± 14.0% vs. ^129^Xe MRI VDP_T_ = 21.0 ± 5.2%, *p* = 0.81). Using the k-means method, Bland-Altman analysis (Figure 2D) indicated a similar 1.6% bias (95% limit of agreement: −10.4% to 7.2%) for higher VDP_K_ measured by Technegas SPECT (Technegas SPECT VDP_K_ = 9.4 ± 9.4% vs. ^129^Xe MRI VDP_K_ = 7.8 ± 10.0%, *p* = 0.02).

Univariate relationships of Technegas SPECT VDP and ^129^Xe MRI VDP with age, pack-year smoking history, spirometry and DL_CO_ are summarized in Table 2. Using the threshold method, Technegas SPECT VDP_T_ and ^129^Xe MRI VDP_T_ were negatively correlated with DL_CO_%_pred_ (r = −0.61, *p* < 0.0001; and r = −0.37, *p* = 0.02) and FEV_1_/FVC (r = −0.38, *p* = 0.01; and r = −0.43, *p* = 0.005). ^129^Xe MRI VDP_T_, but not Technegas SPECT VDP_T_, was correlated with FEV_1_%_pred_ (r = −0.55, *p* = 0.0002). Using the k-means method, Technegas SPECT VDP_K_ and ^129^Xe MRI VDP_K_ were negatively correlated with DL_CO_%_pred_ (r = −0.52, *p* = 0.0005; and r = −0.68, *p* < 0.0001). ^129^Xe MRI VDP_K_, but not Technegas SPECT VDP_K_, was correlated with pack-year smoking history (r = 0.56, *p* = 0.0002), FEV_1_%_pred_ (r = −0.35, *p* = 0.03) and FEV_1_/FVC (r = −0.46, *p* = 0.002). For both modalities, VDP_T_ and VDP_K_ were not different for participants classified by tumor stage or tumor size (Supplementary Table S2). Additionally, for both modalities, VDP_T_ and VDP_K_ of the ipsilateral lung (lung with tumor) was not different than the VDP_T_ and VDP_K_ of the contralateral lung (lung without tumor) (Supplementary Figure S1).

**TABLE 2:** Univariate relationships of Technegas SPECT VDP and ^129^Xe MRI VDP with participant demographics and clinical characteristics.

	Technegas SPECT				^129^Xe MRI			
	VDP_T_, %*		VDP_K_, %		VDP_T_, %		VDP_K_, %*	
	r	*p*	r	*p*	r	*p*	r	*p*
Age years	0.02	0.91	0.04	0.80	−0.24	0.13	0.28	0.07
Pack-year smoking history	0.25	0.10	0.21	0.18	−0.02	0.89	0.56	**0.0002**
FEV_1_%_pred_	−0.26	0.12	−0.26	0.09	−0.55	**0.0002**	−0.35	**0.03**
FVC%_pred_	−0.08	0.60	−0.16	0.31	−0.26	0.10	−0.19	0.24
FEV_1_/FVC, %	−0.38	**0.01**	−0.28	0.07	−0.43	**0.005**	−0.46	**0.002**
DL_CO_%_pred_	−0.61	**<0.0001**	−0.52	**0.0005**	−0.37	**0.02**	−0.68	**<0.0001**

Relationships were evaluated with Pearson correlation coefficients for parametric data and Spearman’s correlation coefficients for non-parametric data. VDP = ventilation defect percent; VDP_T_ = VDP, determined by thresholding method; VDP_K_ = VDP, determined by k-means method; FEV_1_ = forced expiratory volume in one second; FVC = forced vital capacity; DL_CO_ = diffusion capacity for carbon monoxide. *Thresholding (VDP_T_) and k-means clustering (VDP_K_) methods previously optimized and validated for Technegas SPECT, and ^129^Xe MRI, respectively. Bold values denote statistical significance at the *p* <0.05 level.

**FIGURE 3:**
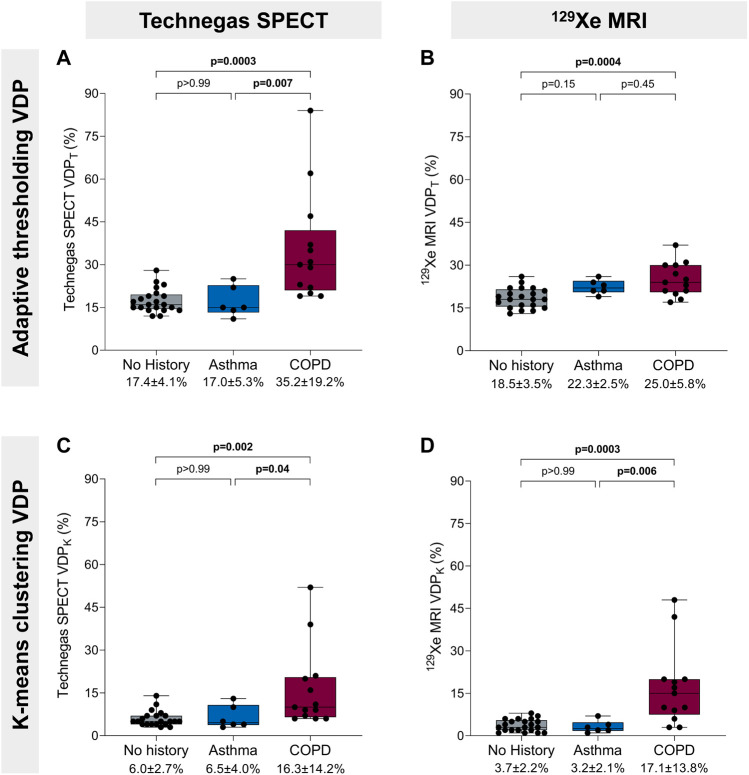
Ventilation defect percent and history of obstructive lung disease. **(A)** Technegas SPECT VDP_T_* was higher in participants with COPD than participants with asthma (35.2% ± 19.2% vs. 17.0 ± 5.3, *p* = 0.007) and those with no history of lung disease (35.2% ± 19.2% vs. 17.4 ± 4.1, *p* = 0.0003). **(B)** ^129^Xe MRI VDP_T_ was higher for participants with COPD than those with no history of lung disease (25.0% ± 5.8% vs. 18.5% ± 3.5%, *p* = 0.0004). **(C)** Technegas SPECT VDP_K_ was significantly higher for participants with COPD than those with asthma (16.3% ± 14.2% vs. 6.5% ± 4.0%, *p* = 0.04) and no history of lung disease (16.3% ± 14.2% vs. 6.0% ± 2.7%, *p* = 0.002). **(D)** ^129^Xe MRI VDP_K_* was higher in participants with COPD than participants with asthma (17.1% ± 13.8% vs. 3.2% ± 2.1%, *p* = 0.006) and those with no history of lung disease (17.1% ± 13.8% vs. 3.7% ± 2.2%, *p* = 0.0003). Box plots show minimum, first quartile, median, third quartile, and maximum VDP with individual values for all participants superimposed on the plot. Difference between groups was determined using one-way ANOVA with Tukey’s multiple comparisons test or Kruskal Wallis with Dunn’s multiple comparisons test. *Thresholding (VDP_T_) and k-means clustering (VDP_K_) methods previously optimized and validated for Technegas SPECT and ^129^Xe MRI, respectively.

Figure 3 summarizes VDP for 21 (51%) participants with no concomitant obstructive lung disease, 6 (15%) with a history of asthma, and 13 (32%) with COPD. The one (2%) participant with interstitial lung disease was excluded from this cross-sectional comparison. Using the threshold method (Figures 3A, B), Technegas SPECT VDP_T_ and ^129^Xe MRI VDP_T_ were significantly higher for participants with COPD than with those with no history of lung disease (*p* = 0.0003 and *p* = 0.0004). Technegas SPECT VDP_T_, but not ^129^Xe MRI VDP_T,_ was significantly higher for participants with COPD than with those with asthma (*p* = 0.007 and *p* = 0.45). There was no difference in Technegas SPECT VDP_T_ or ^129^Xe MRI VDP_T_ between participants with asthma and those with no history of lung disease (*p* > 0.99 and *p* = 0.15). Using the k-means method (Figures 3C, D), Technegas SPECT VDP_K_ and ^129^Xe MRI VDP_K_ were significantly higher for participants with COPD than with those with asthma (*p* = 0.04 and *p* = 0.006) and no history of lung disease (*p* = 0.002 and *p* = 0.0003). There was no difference in Technegas SPECT VDP_K_ or ^129^Xe MRI VDP_K_ between participants with asthma and those with no history of lung disease (*p* > 0.99 and *p* > 0.99).

## 4 Discussion

We prospectively compared ventilation defects assessed by same-day Technegas SPECT and ^129^Xe MRI in 41 patients scheduled to undergo first-time lung cancer resection, a subset of whom had concomitant asthma or COPD. We report that ventilation defects quantified by Technegas SPECT and ^129^Xe MRI VDP (determined using both adaptive thresholding and k-means clustering segmentation methods) were 1) correlated with one another, 2) similarly correlated with standard measures of airflow limitation (FEV_1_/FVC) and diffusing capacity (DL_CO_%_pred_), and 3) significantly higher for participants with COPD than those with asthma and no history of obstructive lung disease.

Many segmentation methods have been developed and optimized to quantify ventilation defects as the VDP, including linear binning, thresholding, and k-means clustering. In this study, VDP was determined for both Technegas SPECT and ^129^Xe MRI using adaptive thresholding and k-means segmentation methods. The basis for this decision was that the adaptive thresholding quantification method has been previously optimized and validated for Technegas SPECT by Farrow et al., (2012), and the k-means method for ^129^Xe MRI by Kirby et al., (2012). Using both segmentation approaches, we observed that the burden of ventilation defects quantified by Technegas SPECT and ^129^Xe MRI VDP acquired on the same day were correlated. While this is the first comparison of ventilation defects assessed by Technegas SPECT and ^129^Xe MRI, our observations are consistent with previous investigations demonstrating the comparability of ventilation assessed by SPECT and MRI when utilizing alternative ventilation agents. Stavngaard and colleagues previously reported a good correlation between ^81m^Kr SPECT and ^3^He MRI for both visual and quantitative assessments of ventilation defect scores in a cohort of 23 COPD and 9 healthy participants (Stavngaard et al., 2005). In a smaller study of 11 COPD patients, Doganay et al. demonstrated a good correlation between ^99m^Tc-DTPA SPECT and ^129^Xe MRI relative lobar percentage ventilation (Doganay et al., 2019). For ventilation SPECT, international guidelines now recommend Technegas as the preferred ventilation agent in patients with obstructive lung disease (Roach et al., 2013; Bajc et al., 2019) limiting the clinical relevance of previous comparisons that used ^81m^Kr and ^99m^Tc-DTPA. Additionally, for hyperpolarized gas ventilation MRI, ^129^Xe gas is now preferred over ^3^He gas due to its greater availability, lower cost, and higher solubility that permits dissolved-phase imaging (Niedbalski et al., 2021).

In most participants, visual assessment showed spatial agreement between focal ventilation defects observed by both modalities. However, as highlighted in Figure 1 by the blue arrows, some discordance was also observed. We also report a mean bias, 2.0% and 1.6%, towards higher VDP measured by Technegas SPECT than ^129^Xe MRI, which was observed using the adaptive threshold and k-means method, respectively. This inter-modality bias and lack of absolute agreement in ventilation defects were not unexpected and may be explained by several factors. First, fundamental differences in the physical properties of the ventilation agents may contribute to differences in lung distribution. Technegas is an ultrafine aerosolized particle (0.005–0.2 μm (Lemb et al., 1993)) whose distribution in the lungs, unlike that of ^129^Xe gas, is impacted by aerosol deposition mechanics. While Technegas behaves in a gas-like manner, permitting peripheral penetration and alveolar deposition (Bajc et al., 2019), it has been previously shown to aggregate at sites of severe obstruction leading to “hot-spots” (De Nijs et al., 2021). Shown in Figure 1D, we observed this effect in a 65-year-old male with severe COPD (FEV_1_ = 28%_pred_, FEV_1_/FVC = 31%). Bilateral hotspots are observed on Technegas SPECT in the left and right main bronchi. Greater ventilation is observed distal to the right main bronchi hotspot by ^129^Xe MRI than Technegas SPECT. Second, the different acquisition conditions and spatial resolutions between modalities must be considered. Technegas SPECT is acquired during 15 min of tidal breathing, while ^129^Xe MRI is acquired during a 10 s breath hold at functional residual capacity plus 1 L. As a result, respiratory and cardiac motion have greater influence on ventilation assessed by SPECT, contributing to blurring and fewer counts at the lung borders. Additionally, lung inflation during imaging affects ventilation defects, with increased ventilation defects observed at lower levels of lung inflation (Hughes et al., 2019). As SPECT is on average acquired at a lower lung inflation (average over tidal volume) than ^129^Xe MRI (functional residual capacity plus 1 L), higher VDP is expected. Taken together, the aforementioned factors likely account for the higher VDP quantified by Technegas SPECT and the spatial discordances in focal ventilation defects that were observed upon visual inspection.

It is important to emphasize that this study did not intend to determine the optimal quantification approach for each modality, rather to determine if there was correlation and some equivalency between the two modalities using established quantification practices for each modality that are implemented in the literature. There are reasons why each modality may best be served by different segmentation methods, which is beyond the scope of this article. However, we do note that for both modalities, the adaptive threshold method resulted in significantly higher VDPs compared to the k-means clustering method, irrespective of history of obstructive lung disease. In the subgroup of patients with no history of lung disease, the majority of whom had well-ventilated lungs by visual inspection, the VDPs determined using the k-means method were much closer to zero than the threshold method (Technegas SPECT: VDP_K_ = 6.0% vs. VDP_T_ = 17.4%; ^129^Xe MRI: VDP_K_ = 3.7% vs. VDP_T_ = 18.5%), which better reflects what the images show (e.g., Figure 1A). We also noticed that when the VDP was determined using the threshold method (but not the k-means method), the bias towards higher Technegas SPECT VDP_T_ increased with VDP_T_, or greater airflow limitation (Figure 2C). This bias seems to be driven largely by a subset of patients with COPD in whom the Technegas SPECT VDP_T_ was considerably higher than the ^129^Xe MRI VDP_T_. Taken together, investigation of these cases reveals that the adaptive threshold classifies hypo-ventilated (or low ventilated) voxels as defect, leading to a significantly higher VDP_T_, which can be misleading when interpreted in absolute terms. This effect, in combination with severe obstruction leading to “hot-spots,” likely explains the exceptionally high Technegas SPECT VDP_T_ reported for two patients with COPD (62% and 84%).

Ventilation defect burden quantified by Technegas SPECT VDP_T_ and ^129^Xe MRI VDP_K_ (VDPs determined using modality-specific approach) were similarly correlated to standard measures of airflow limitation (FEV_1_/FVC) and diffusing capacity (DL_CO_). For both modalities, the correlation with DL_CO_ was stronger than with FEV_1_/FVC. One explanation for this may be that DL_CO_ is a direct measurement of the capacity of communicating lung volume to transfer gas from inhaled air to the bloodstream, whereas FEV_1_/FVC is an indirect composite marker of the presence of airway obstruction with forced exhalation. Interestingly, ^129^Xe MRI VDP_K_ but not SPECT VDP_T_, was correlated with pack-year smoking history and FEV_1_. The reason for this discrepancy is unclear. Consistent with these relationships, we also observed greater ventilation defect burden quantified by both modalities in participants with COPD compared to those with asthma and no history of obstructive disease. However, VDP assessed by both modalities was not higher in patients who reported a history of asthma compared to those without any known history of obstructive disease. This result may be considered unexpected as abnormal ventilation is a characteristic feature of asthma. We do note that the VDPs in our cohort of asthmatics are similar to what has been previously reported by others, using ^129^Xe MRI VDP_K_ (Eddy et al., 2022) and Technegas SPECT VDP_T_ (Farrow et al., 2012; Farrow et al., 2017), respectively. When interpreting this result, it should be considered that most of our cohort of patients without any known history of obstructive lung disease were current smokers (4 of 21, 19%) or past smokers (10 of 21, 48%), 36% of whom had an FEV_1_/FVC less than 0.70 and 7% had a DL_CO_ less than 80%_pred_. As demonstrated by participant B in Figure 1, such patients may have subclinical or undiagnosed airways disease contributing to ventilation defects and increased VDP.

There are limitations to our study that should be considered. First, we evaluated a convenient sample of patients scheduled for lung cancer resection, in whom tumor burden may have influenced segmentation of the thoracic cavity and VDP quantification. However, in our cohort, tumor size was not associated with whole-lung VDP, and ipsilateral and contralateral VDP were not different (data provided online, Supplementary Figure S1; Table S2). These observations indicate that tumor burden did not significantly contribute to VDP assessed by either modality, which is not surprising given the small tumor sizes in our cohort (≤3 cm in 46%, >3 to ≤5 cm in 27%, >5 to ≤7 cm in 10%, and >7 cm in 7% of participants). Second, we did not quantitatively evaluate the spatial agreement of ventilation defects observed by Technegas SPECT and ^129^Xe MRI. Such analysis is highly dependent on accurate registration and anatomical alignment of Technegas SPECT and ^129^Xe MRI datasets, which was challenging due to differences in voxel size, acquisition conditions (tidal breathing vs. breath hold) and lung volume. Finally, our quantitative analysis distilled regional and voxel-wise measurements of ventilation down to a single whole-lung value, in this case the VDP. VDP is a binary whole-lung measurement that fails to characterize much of the information offered by ventilation imaging modalities. Furthermore, as our analysis demonstrates, different segmentation schemes yield different VDPs in the same individual.

In summary, using the current ideal contrast agents for ventilation SPECT and MRI, we imaged patients with and without obstructive lung disease prior to lung cancer resection to quantify and compare ventilation defects observed by both modalities. We report that the burden of ventilation defects quantified by Technegas SPECT and ^129^Xe MRI are correlated and increased in participants with COPD. Our observations indicate that, despite substantial differences between the imaging modalities, assessment of ventilation defects using established quantification practices for Technegas SPECT and ^129^Xe MRI are comparable, provided the same quantification approach is used. Future work is required to determine if superior and comparable improvements in patient outcomes are achieved by integrating ventilation assessment with Technegas SPECT and ^129^Xe MRI into the clinical management of lung diseases and potential improvement in outcomes post-resection. For now, based on our findings, the selection of ventilation imaging modality can be guided by local availability and regulatory approval, contraindications, and concern of radiation burden.

## Data Availability

Data sharing requests will be considered from researchers that submit a proposal and an appropriate statistical analysis and dissemination plan. Data would be shared via a secure data access system by request to the corresponding author.
