# Supplementary material for: Comparison of ventilation defects quantified by Technegas SPECT and hyperpolarized 129Xe MRI
Source: Front Physiol. 2023 Apr 28;14:1133334. doi: 10.3389/fphys.2023.1133334 (PMC10206636; doi:10.3389/fphys.2023.1133334)
Supplement: Supplementary file 1 [file Table1.DOCX]

Supplementary Material

## Supplementary Figures and Tables

**Table S1.** Dosing and measurements of image quality

|  | **All participants**  **(n=41)** |
| --- | --- |
| **Technegas SPECT** |  |
| ***Dose*** |  |
| Activity of ^99m^Tc sodium pertechnetate loaded into crucible (MBq) | 581 [400-815] |
| Number of breaths | 6 [2-15] |
| ***Image quality*** |  |
| Count rate from lungs on γ-camera, posterior (counts/second)^†^ | 2300 [930-4350] |
| Count rate from lungs on γ-camera, anterior (counts/second)^†^ | 2500 [720-4520] |
| **^129^Xe MRI** |  |
| ***Dose*** |  |
| Volume of ^129^Xe delivered (mL) | 600 [500-730] |
| Polarization (%) | 10 [9-34] |
| Dose equivalent volume (mL)^‡^ | 54 [46-146] |
| ***Image quality*** |  |
| Signal-to-noise ratio^*^ | 37 [14-92] |

Values median [minimum-maximum].

^†^A lower limit of 1500 counts/second is recommended.

^‡^A lower limit of 50mL is recommended.

^*^Signal-to-noise ratio (SNR) for ^129^Xe MRI was calculated as the mean voxel value within four representative regions of interest (ROI) within the ventilated lung divided by the standard deviation of the voxel values for noise inside four representative ROIs of the same size outside of the thoracic cavity (i.e., background).

**
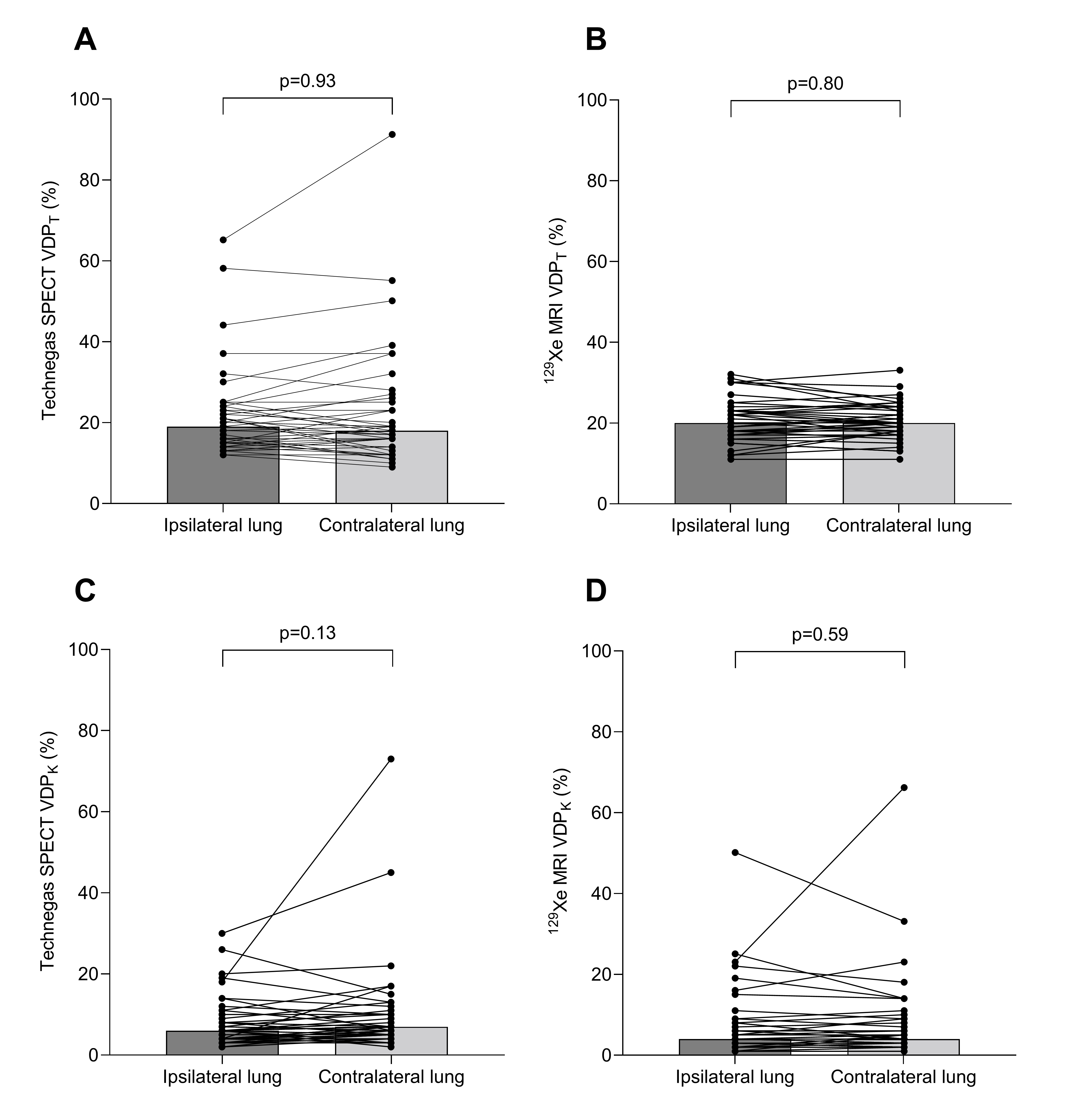
**

**Figure S1.** **Comparison of ipsilateral and contralateral lung ventilation defect percent (VDP) assessed by Technegas SPECT and ^129^Xe MRI.**

**(A)** Technegas SPECT VDP_T_ of the ipsilateral (19% [12-65%]) and contralateral (18% [9-91%]) lung was not different (p=0.93).

**(B)** ^129^Xe MRI VDP_T_ of the ipsilateral (20% [11-32%]) and contralateral (20% [11-33%]) lung was not different (p=0.80).

**(C)** Technegas SPECT VDP_K_ of the ipsilateral (6% [2-30%]) and contralateral (7% [2-73%]) lung was not different (p=0.13).

**(D)** ^129^Xe MRI VDP_K_ of the ipsilateral (4% [1-50%]) and contralateral (4% 1-66%]) lung was not different (p=0.59).

VDP_T_=VDP determined by thresholding method; VDP_K_=VDP determined by k-means method. Bars represent median with paired ipsilateral and contralateral lung values for all participants superimposed on the plot. Difference between ipsilateral and contralateral lung VDP was determined using paired t-test (parametric data) or Wilcoxon test (non-parametric data)

**Table S2.** Ventilation defect percent by tumor stage and tumor size as per TNM-staging 8th edition.

|  | **Tumor Stage**^†^ | | | |  | **Tumor Size**^†^ | | | |
| --- | --- | --- | --- | --- | --- | --- | --- | --- | --- |
|  | I  (n=25) | II  (n=7) | III&IV  (n=5) | p-  value* |  | T1  (n=19) | T2  (n=11) | T3&T4  (n=7) | p-value* |
| **Technegas SPECT** | | | | | | | | | |
| VDP_T_ | 19  [12-62] | 17  [14-35] | 18  [11-84] | 0.76 |  | 22  [14-62] | 16  [12-24] | 18  [11-84] | 0.19 |
| VDP_K_ | 6  [3-39] | 6  [4-16] | 8  [3-52] | 0.91 |  | 6  [3-39] | 5  [3-11] | 11  [3-52] | 0.20 |
| **^129^Xe MRI** | | | | | | | | | |
| VDP_T_ | 21  [14-30] | 18  [13-24] | 22  [16-37] | 0.16 |  | 21  [14-30] | 20  [15-23] | 23  [13-37] | 0.35 |
| VDP_K_ | 4  [1-42] | 4  [2-20] | 6  [1-48] | 0.91 |  | 5  [1-42] | 2  [1-6] | 6  [1-48] | 0.05 |

Values are median [minimum-maximum]. VDP_T_=VDP determined by thresholding method; VDP_K_=VDP determined by k-means method. ^†^As per TNM-staging 8^th^ edition. *Significance of difference between groups was determined using Kruskal Wallis with Dunn’s multiple comparisons test.
